# Clinical Implications of Post-Earthquake Environmental Exposures in Children with Allergic Diseases

**DOI:** 10.3390/jcm15082875

**Published:** 2026-04-10

**Authors:** Fatih Kaplan, Bilge Kurnaz Kaplan, Emrullah Arıkanoğlu, Abdulgani Gülyüz

**Affiliations:** 1 Department of Pediatric Allergy and Immunology, Malatya Training and Research Hospital, 44000 Malatya, Türkiye; fatih.kaplan@inonu.edu.tr; 2 Department of Otorhinolaryngology (ENT), Malatya Training and Research Hospital, 44000 Malatya, Türkiye; bilge.kurnazkaplan@inonu.edu.tr; 3 Department of Pediatrics, Malatya Training and Research Hospital, 44000 Malatya, Türkiye; emrullah_arikanoglu@hotmail.com; 4 Department of Pediatrics, Faculty of Medicine, Malatya Turgut Özal University, 44210 Malatya, Türkiye

**Keywords:** earthquake, pediatric allergic diseases, asthma, environmental exposure, particulate matter, temporary housing, demolition dust, disease control

## Abstract

**Highlights:**

**What are the main findings?**

- Post-earthquake environmental exposures were associated with worsening disease control in children with allergic diseases.
- Prolonged residence in temporary housing and exposure to demolition-related dust were independently associated with clinical deterioration, particularly among children with asthma.

**What are the implications of the main findings?**

- Children with chronic allergic diseases should be considered a vulnerable population in post-disaster settings.
- Dust-control measures, air-quality monitoring, and improved living conditions in temporary housing may help reduce post-disaster worsening of allergic diseases.

**Abstract:**

**Background/Objectives**: Environmental changes following large-scale natural disasters may influence the clinical course of chronic diseases. However, the impact of post-earthquake environmental exposures on pediatric allergic diseases remains insufficiently studied. To evaluate the association between post-earthquake environmental exposures and disease control in children with allergic diseases. **Methods**: This retrospective longitudinal cohort study included 528 children with previously diagnosed asthma, allergic rhinitis, or atopic dermatitis who were followed in a tertiary pediatric allergy center in Malatya, Türkiye. Clinical assessments performed before the 6 February 2023 Kahramanmaraş earthquakes (T0) were compared with follow-up evaluations conducted 6–12 months after the earthquake (T1). Environmental exposures assessed during the post-earthquake period included prolonged residence in temporary housing, demolition-related dust exposure, and elevated ambient particulate matter levels. Clinical deterioration was defined using disease-specific indicators (decline in ACT/cACT score or treatment step escalation for asthma, increase in TNSS for allergic rhinitis, and increase in SCORAD for atopic dermatitis). Multivariable logistic and linear regression models were used to evaluate associations between environmental exposures and clinical outcomes. **Results**: Clinical deterioration was observed in 219 children (41.5%). Prolonged residence in temporary housing for ≥6 months (aOR 2.1, 95% CI 1.2–3.9, *p* = 0.01) and exposure to demolition-related dust (aOR 1.9, 95% CI 1.1–3.5, *p* = 0.02) were independently associated with clinical deterioration. Among children with asthma, both prolonged temporary housing (adjusted β −1.84, *p* = 0.002) and demolition-related dust exposure (adjusted β −1.39, *p* = 0.018) were associated with worsening asthma control. **Conclusions**: Post-earthquake environmental exposures, particularly prolonged residence in temporary housing and demolition-related dust exposure, were associated with worsening control of pediatric allergic diseases. These findings highlight the importance of environmental health considerations in disaster response and long-term management of children with chronic allergic conditions.

## 1. Introduction

The frequency of natural disasters and the number of people affected by them have increased markedly in recent years, making these events a significant global public health concern [1,2]. Earthquakes and other large-scale disasters are often primarily addressed in terms of acute trauma, infectious diseases, and the immediate need for emergency medical services [3]. However, the health consequences of disasters extend beyond the acute phase. Environmental changes, deteriorating living conditions, and disruptions in access to healthcare during the post-disaster period may adversely affect the management of chronic diseases [4,5]. Therefore, disaster response strategies should address not only acute care but also the continuity of chronic disease management.

Children represent one of the most vulnerable populations in post-disaster environments due to their ongoing physiological development and increased susceptibility to environmental exposures [6]. Among the most common chronic diseases in childhood are asthma, allergic rhinitis, and atopic dermatitis, the prevalence of which has been steadily increasing worldwide [7,8]. Asthma, in particular, is the most common chronic respiratory disease in children and affects millions globally [9]. The clinical course of pediatric allergic diseases is influenced not only by genetic predisposition but also by environmental and socioeconomic factors, including air quality, allergen exposure, living conditions, and access to healthcare [10].

Air pollution, particularly exposure to particulate matter, is a well-established environmental determinant of allergic disease exacerbations. Fine particles such as PM10 and PM2.5 can increase airway inflammation and have been associated with asthma exacerbations, worsening respiratory symptoms, and increased healthcare utilization [11]. Following large-scale earthquakes, demolition and debris removal activities may substantially increase airborne particulate matter concentrations. In addition, temporary housing conditions, crowding, and changes in indoor environmental exposures during the post-disaster period may further complicate the control of allergic diseases.

The Kahramanmaraş-centered earthquakes that occurred on 6 February 2023 resulted in substantial environmental and social changes across a wide geographical region of Türkiye. The aim of this study was to evaluate the association between post-earthquake environmental exposures—including demolition-related dust, increased particulate matter levels, and prolonged residence in temporary housing—and disease control among children previously diagnosed with asthma, allergic rhinitis, or atopic dermatitis. The study also aimed to identify environmental factors that may influence the management of pediatric allergic diseases in post-disaster settings. We hypothesized that post-earthquake environmental exposures, particularly prolonged temporary housing and demolition-related dust, would be associated with worsening disease control in children with allergic diseases.

## 2. Materials and Methods

### 2.1. Study Design and Population

This study was designed as a retrospective longitudinal cohort study conducted at a tertiary pediatric center in Malatya following the Kahramanmaraş-centered earthquakes that occurred on 6 February 2023. The aim of the study was to evaluate the effects of post-earthquake environmental exposures—particularly temporary housing conditions, demolition-related dust exposure, and increased particulate matter levels—on disease control and clinical course in children previously diagnosed with asthma, allergic rhinitis, or atopic dermatitis before the earthquake. Pre-earthquake and post-earthquake clinical assessments were compared within the same patient population, allowing evaluation of the potential impact of post-disaster environmental changes on the control of pediatric allergic diseases.

The study included pediatric patients followed in the pediatric allergy outpatient clinic of the participating center in Malatya who had been diagnosed with asthma, allergic rhinitis, or atopic dermatitis before the earthquake. Although allergic diseases may coexist in the same patient, for analytical purposes, patients were classified according to the primary allergic diagnosis documented in the medical records. Accordingly, patients were categorized into three main groups: asthma, allergic rhinitis, and atopic dermatitis. In cases where more than one allergic disease was present, the clinically dominant condition was considered the primary diagnosis. Each patient was assigned to only one diagnostic group, and the disease groups were treated as mutually exclusive categories for comparison purposes. Coexisting allergic diseases were also recorded and included as covariates in disease-specific statistical analyses.

Patients were included if they were between 0 and 18 years of age; had a diagnosis of asthma, allergic rhinitis, or atopic dermatitis established before the earthquake on 6 February 2023; had at least one outpatient visit within the six months preceding the earthquake; and had at least one follow-up visit between six and twelve months after the earthquake. Patients newly diagnosed after the earthquake and those with incomplete clinical data or insufficient follow-up information were excluded. In the asthma subgroup, patients with excessively severe baseline disease were excluded to prevent distortion of outcome measures. These included patients with two or more exacerbations requiring systemic corticosteroids within the previous six months or a history of hospitalization due to asthma. In the allergic rhinitis and atopic dermatitis groups, baseline disease severity was included in the analyses as an adjusting covariate.

**Figure 1:**
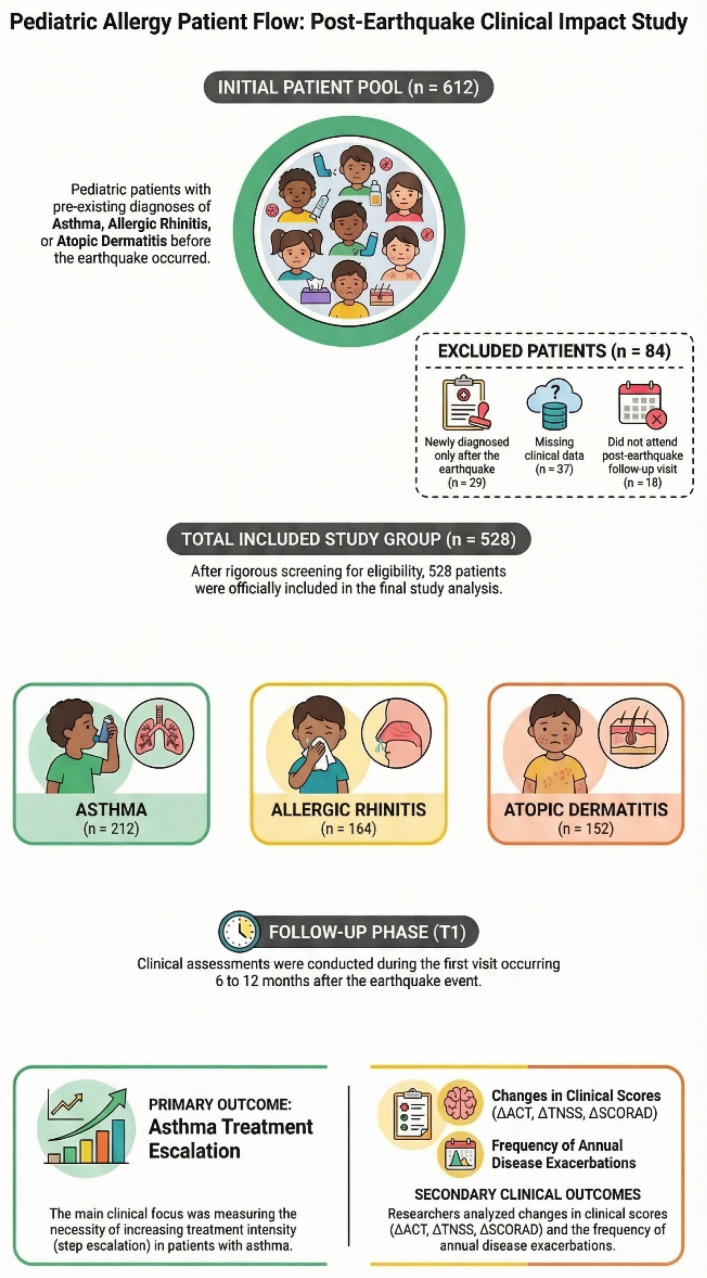
Patient flow diagram.

Two time points were defined in the study. Baseline (T0) was defined as the last outpatient clinic visit before the earthquake within the six months preceding the event. The follow-up visit (T1) was defined as the first scheduled outpatient visit during the post-earthquake period, between six and twelve months after the earthquake. The interval between T0 and T1 was calculated in days and included as an adjustment variable in statistical analyses. The patient selection and inclusion process is illustrated in Figure 1.

### 2.2. Baseline Assessment

During the T0 visit, demographic and clinical variables, including age, sex, disease duration, and baseline treatment step, were recorded. Disease control was evaluated using validated clinical instruments. Asthma control was assessed using the Asthma Control Test (ACT) or Childhood Asthma Control Test (cACT), along with GINA. treatment steps. Allergic rhinitis severity was evaluated using the Total Nasal Symptom Score (TNSS), and atopic dermatitis severity was assessed using the SCORAD index. Baseline disease severity was incorporated into statistical models using baseline ACT/cACT scores and GINA treatment steps for asthma, baseline TNSSs for allergic rhinitis, and baseline SCORAD scores for atopic dermatitis [12,13,14].

Additional environmental and clinical variables recorded at baseline included the number of exacerbations in the previous six months, passive tobacco smoke exposure, indoor dampness or mold, and household crowding.

Skin prick test (SPT) results were obtained from patient records for individuals who had undergone testing as part of routine clinical evaluation using standardized allergen extracts. Tests were performed and interpreted according to established clinical guidelines. Since SPT data were available for 376 patients, allergen sensitization rates were calculated based on the number of patients who had undergone testing.

### 2.3. Assessment of Environmental Exposures

The duration of residence in temporary housing after the earthquake, including tents or container settlements, was obtained from patient records and follow-up forms. Housing duration was categorized into three groups: no temporary housing, less than six months, and six months or longer. In the primary analyses, residence in temporary housing for six months or longer was considered the main exposure variable.

Environmental dust exposure related to demolition and debris removal activities was assessed using structured caregiver interviews and information recorded in clinical follow-up forms. Dust exposure was defined as the presence of at least one of the following indicators: ongoing demolition activities near the residence, debris removal operations in the surrounding area, visible dust accumulation around the home or school, or recurrent dense dust clouds. Exposure around home and school environments was evaluated separately, and the presence of exposure in either location was considered positive exposure.

Ambient air pollution data were obtained from official monitoring stations belonging to the Turkish National Air Quality Monitoring Network [15]. Particulate matter levels were evaluated for two periods: the pre-earthquake period (February 2022–January 2023) and the post-earthquake period (February 2023–December 2024). Areas with high particulate matter exposure were defined as residential districts with mean PM10 or PM2.5 levels above the citywide median during the post-earthquake monitoring period.

Socioeconomic status was assessed using indicators including type of health insurance, parental education level, parental employment status, and household size. These variables were scored to generate a three-level socioeconomic classification: low, middle, and high. Indoor environmental factors analyzed in the study included passive tobacco smoke exposure, indoor dampness or mold, and household crowding, defined as two or more persons per room.

### 2.4. Outcome Measures

The primary outcome of the study was defined as clinical deterioration. Clinical deterioration was defined as worsening in at least one disease-specific indicator: a decline in ACT/cACT score or escalation of treatment step in asthma, an increase in TNSS in allergic rhinitis, or an increase in SCORAD score in atopic dermatitis. This composite definition allowed joint analysis of different allergic diseases within the same cohort.

Secondary outcomes included change in asthma control measured by ΔACT/cACT, change in allergic rhinitis symptoms measured by ΔTNSS, change in atopic dermatitis severity measured by ΔSCORAD, annual exacerbation frequency, and change in treatment step among children with asthma. Change scores were calculated as follows: ΔACT = ACT(T1) − ACT(T0), ΔTNSS = TNSS(T1) − TNSS(T0), and ΔSCORAD = SCORAD(T1) − SCORAD(T0).

### 2.5. Statistical Analysis

Statistical analyses were performed using SPSS version 26.0 (IBM Corp., Armonk, NY, USA). Continuous variables were reported as mean ± standard deviation or as median with interquartile range, where appropriate. Categorical variables were expressed as numbers and percentages. Multivariable logistic regression models were constructed to evaluate the associations between environmental exposures and clinical deterioration, and the results were reported as adjusted odds ratios with 95% confidence intervals. Multivariable linear regression models were used for continuous outcome variables, including ΔACT, ΔTNSS, and ΔSCORAD. All multivariable models were adjusted for potential confounding variables, including age, sex, socioeconomic status, passive tobacco smoke exposure, indoor dampness or mold, and baseline disease severity. A *p*-value of less than 0.05 was considered statistically significant.

## 3. Results

A total of 528 children with previously diagnosed allergic diseases were included in the study. Among them, 212 (40.2%) had asthma, 164 (31.1%) had allergic rhinitis, and 152 (28.8%) had atopic dermatitis. The mean age of the overall cohort was 8.7 ± 4.2 years, and 302 patients (57.2%) were male. The mean disease duration was 3.1 ± 2.2 years. Passive tobacco smoke exposure was reported in 211 children (39.9%), while 146 children (27.6%) lived in households with indoor dampness or mold. Baseline demographic and clinical characteristics of the study population, stratified by allergic disease subgroup, are presented in Table 1.

Environmental exposure characteristics during the post-earthquake period are summarized in Table 2. Overall, 187 children (35.4%) lived in temporary housing for ≥6 months, while 118 children (22.3%) stayed in temporary settlements for less than 6 months. The remaining 223 children (42.2%) continued to reside in permanent housing. Exposure to demolition-related dust in the residential or school environment was reported in 241 children (45.6%). In addition, 198 children (37.5%) resided in areas classified as having elevated ambient particulate matter levels during the post-earthquake period (Table 2).

Changes in disease-specific clinical outcomes between baseline (T0) and follow-up (T1) are presented in Table 3. Among children with asthma, the mean ACT/cACT score decreased from 18.2 ± 4.6 at baseline to 16.5 ± 6.2 at follow-up, corresponding to a mean change of −1.7 ± 4.8 (*p* = 0.02). In the allergic rhinitis subgroup, the TNSS increased from 3.4 ± 1.7 to 3.9 ± 2.8, representing a mean change of +0.5 ± 2.1 (*p* = 0.06). Among children with atopic dermatitis, the SCORAD index increased from 35.8 ± 19.8 at baseline to 43.1 ± 24.5 at follow-up, corresponding to a mean increase of 7.3 ± 18.4 (*p* = 0.03). Across the entire cohort, the annual exacerbation frequency increased from 0.9 ± 1.2 to 1.8 ± 2.1 episodes per year (*p* < 0.001) (Table 3).

Using the predefined composite definition of disease-specific worsening, clinical deterioration between baseline (T0) and follow-up (T1) was observed in 219 children (41.5%). To identify environmental and clinical factors associated with clinical deterioration, a multivariable logistic regression model was constructed. Prolonged residence in temporary housing for ≥6 months was independently associated with an increased likelihood of clinical deterioration (aOR 2.1, 95% CI 1.2–3.9, *p* = 0.01). Exposure to demolition-related dust was also significantly associated with deterioration (aOR 1.9, 95% CI 1.1–3.5, *p* = 0.02). Passive tobacco smoke exposure remained an independent predictor (aOR 1.7, 95% CI 1.0–2.9, *p* = 0.04). Residence in areas with elevated particulate matter levels showed a positive but non-significant association with clinical deterioration (aOR 1.6, 95% CI 0.9–2.8, *p* = 0.08). Indoor dampness or mold was not significantly associated with clinical deterioration (aOR 1.4, 95% CI 0.8–2.5, *p* = 0.22) (Table 4).

Among the 212 children with asthma, treatment step escalation according to GINA recommendations occurred in 78 patients (36.8%), while 108 patients (50.9%) had no change in treatment step, and 26 patients (12.3%) experienced treatment step reduction. To further evaluate changes in asthma control, a multivariable linear regression model was performed to assess factors associated with the change in ACT/cACT score. Prolonged residence in temporary housing for ≥6 months was associated with a greater decline in ACT/cACT score (adjusted β −1.84, 95% CI −3.02 to −0.66, *p* = 0.002). Similarly, exposure to demolition-related dust was independently associated with worsening asthma control (adjusted β −1.39, 95% CI −2.54 to −0.24, *p* = 0.018). High particulate matter exposure showed a trend toward worsening ACT/cACT scores but did not reach statistical significance (adjusted β −1.12, 95% CI −2.31 to 0.07, *p* = 0.065). Passive tobacco smoke exposure and indoor dampness were not statistically significant predictors of ACT/cACT score change after adjustment (Table 5).

Ambient air quality monitoring data demonstrated an increase in particulate matter levels in Malatya during the post-earthquake period. The mean PM10 concentration increased from 30.1 µg/m^3^ before the earthquake to 107.6 µg/m^3^ during the post-earthquake period. Similarly, PM2.5 levels increased from 16.8 µg/m^3^ to 47.6 µg/m^3^ (Table 6).

Subgroup analyses exploring potential effect modification are presented in Supplementary Table S1. The association between demolition-related dust exposure and clinical deterioration was stronger among children with more severe baseline disease (OR 2.9, 95% CI 1.3–6.7; *p* for interaction = 0.03). No significant interaction was observed between temporary housing and age group (*p* for interaction = 0.18) (Supplementary Table S1).

## 4. Discussion

In this study, we evaluated the potential impact of environmental conditions emerging after a large-scale earthquake on the clinical course of pediatric allergic diseases. Our findings suggest that prolonged residence in temporary housing and exposure to demolition-related dust may be associated with worsening disease control in children. Environmental disruptions following disasters are increasingly recognized as important determinants of health, particularly in populations vulnerable to environmental exposures, such as children with chronic respiratory diseases [16,17,18].

Air pollution represents one of the most important environmental determinants of respiratory disease worldwide. Numerous studies have demonstrated that exposure to particulate matter is associated with airway inflammation, bronchial hyperresponsiveness, and increased frequency of asthma exacerbations [19,20]. Fine particles such as PM2.5 are capable of penetrating deep into the respiratory tract and triggering inflammatory pathways that worsen asthma control [21]. In addition, long-term exposure to traffic-related air pollution has been associated with an increased risk of developing asthma in children [22]. These findings support the hypothesis that increased particulate exposure in the post-earthquake environment may contribute to worsening disease control among children with allergic diseases.

Large-scale disasters may significantly alter environmental conditions in affected regions. Demolition, debris removal, and reconstruction activities following earthquakes can generate substantial amounts of airborne dust and particulate matter. Mineral dust particles, including silica and other construction-related particles, may act as respiratory irritants and contribute to airway inflammation [23]. Previous studies have also reported increased respiratory symptoms and disease burden following environmental changes related to disasters [24].

In addition to outdoor air pollution, living conditions in temporary settlements may influence respiratory health. Temporary housing environments often involve crowding, reduced ventilation, and increased exposure to indoor pollutants, which may negatively affect individuals with chronic respiratory diseases [25]. Such environmental conditions may contribute to worsening asthma control, particularly among children with underlying airway hyperreactivity.

Environmental smoke exposure is another factor known to worsen respiratory disease. Studies examining wildfire smoke exposure have demonstrated increased respiratory symptoms, exacerbations, and healthcare utilization among individuals exposed to particulate pollution [26]. These findings further support the role of airborne particulate exposure as an important trigger for worsening respiratory disease.

Air pollution may also influence allergic diseases beyond the respiratory system. Emerging evidence suggests that environmental pollutants can contribute to inflammatory skin disorders and may aggravate conditions such as atopic dermatitis [27]. Environmental exposures therefore represent a potential common pathway influencing multiple allergic diseases.

Beyond environmental exposures, psychosocial stress represents another potentially important contributor to the observed worsening of atopic dermatitis in the post-earthquake setting. Previous reports following the Great Hanshin-Awaji Earthquake demonstrated that AD exacerbation in disaster-affected populations was associated not only with environmental changes but also with post-disaster psychological stress. Stress-induced neuroimmune dysregulation can impair epidermal barrier function and amplify cutaneous inflammatory responses, thereby contributing to AD flares independently of allergen or pollutant exposure. In the present study, psychological stress was not systematically assessed, and its potential contribution to the observed deterioration in atopic dermatitis cannot be excluded. Future studies evaluating post-disaster AD should incorporate validated measures of psychosocial stress alongside environmental exposure assessments to disentangle these interacting pathways.

From a broader public health perspective, environmental health risks following disasters remain an important yet often underrecognized determinant of chronic disease outcomes. The global burden of disease attributable to pollution highlights the importance of addressing environmental exposures in vulnerable populations [28]. International disaster health frameworks also emphasize the need to consider long-term environmental and public health consequences following disasters [17,18].

Our findings are consistent with existing literature indicating that environmental exposures play a central role in the clinical course of allergic diseases. Monitoring air quality and reducing exposure to airborne particulate matter may therefore represent important strategies to protect children with chronic allergic conditions following disasters. In this context, international guidelines such as the World Health Organization air quality guidelines emphasize the importance of reducing particulate pollution to protect respiratory health [28].

This study has several strengths. First, both pre-earthquake and post-earthquake clinical assessments were available for the same patient population, allowing within-cohort comparisons of disease control. Second, multiple allergic diseases—including asthma, allergic rhinitis, and atopic dermatitis—were evaluated simultaneously, enabling a broader assessment of environmental impacts on pediatric allergic conditions. Third, environmental exposure variables and clinical indicators were analyzed together using multivariable regression models, allowing adjustment for important confounding factors.

Nevertheless, several limitations should be acknowledged. First, the retrospective observational design limits the ability to establish causal relationships between environmental exposures and clinical outcomes. Second, particulate matter exposure was assessed using regional air quality monitoring data rather than individual exposure measurements. Although this approach is commonly used in environmental epidemiology, it may not fully capture individual variability in exposure patterns [20]. Third, demolition-related dust exposure was based on caregiver-reported environmental conditions and may therefore be subject to recall bias. Fourth, psychosocial stress—a factor previously shown to independently contribute to atopic dermatitis exacerbation in post-disaster settings—was not systematically assessed in the present study, and its potential contribution to the observed clinical deterioration cannot be excluded. Finally, the study was conducted in a single regional center, which may limit the generalizability of the findings to other disaster-affected regions with different environmental conditions.

In conclusion, environmental conditions emerging after large-scale earthquakes may influence the clinical course of pediatric allergic diseases. Increased particulate exposure, demolition-related dust, and prolonged residence in temporary housing may contribute to worsening asthma control in children. These findings highlight the importance of integrating environmental health considerations into disaster response planning and chronic disease management strategies in disaster-affected regions [17,18].

### 4.1. Strengths of the Study

The present study has several notable strengths. First, clinical outcomes were evaluated using both pre-earthquake and post-earthquake data obtained from the same patient population, allowing within-cohort comparisons of disease control before and after a major environmental disruption. Second, the study included multiple pediatric allergic diseases—namely asthma, allergic rhinitis, and atopic dermatitis—enabling a broader assessment of how post-disaster environmental conditions may influence different allergic disorders. Third, both clinical indicators and environmental exposure variables, including the duration of temporary housing, demolition-related dust exposure, and regional particulate matter levels, were analyzed together, providing a more comprehensive evaluation of determinants of disease control in disaster-affected populations. Finally, the relatively large sample size (n = 528) and the use of multivariable regression models adjusting for key confounders such as passive tobacco smoke exposure and indoor dampness strengthened the robustness of the findings.

### 4.2. Limitations

Several limitations should be considered when interpreting the findings of this study. First, the retrospective observational design limits the ability to establish causal relationships between environmental exposures and changes in allergic disease control. Second, particulate matter exposure was assessed using regional air-quality monitoring data rather than individual-level measurements, which may have resulted in exposure misclassification and did not capture individual variability in daily exposure patterns. Third, demolition-related dust exposure was based on caregiver-reported environmental conditions and may therefore be subject to recall bias. Fourth, the study was conducted in a single regional center, which may limit the generalizability of the findings to other disaster-affected settings. Finally, several potentially relevant variables could not be fully evaluated. In particular, psychosocial stress—a factor previously shown to independently contribute to atopic dermatitis exacerbation in post-disaster settings—was not systematically assessed in the present study, and its potential contribution to the observed clinical deterioration cannot be excluded. Similarly, medication adherence and viral respiratory infections could not be adequately captured due to limitations inherent in retrospective clinical records.

In addition, the composite outcome combined disease-specific definitions of deterioration across three different allergic conditions. Although this approach enabled cohort-wide analysis, it may have introduced heterogeneity in outcome classification and should therefore be interpreted with caution.

## 5. Conclusions

This study suggests that environmental conditions emerging after large-scale earthquakes may influence the clinical course of pediatric allergic diseases. Prolonged residence in temporary housing and exposure to demolition-related dust were associated with worsening asthma control and increased treatment escalation in children. These findings indicate that the health consequences of disasters may extend beyond acute injuries and infectious diseases and may also affect the management of chronic conditions such as asthma and other allergic diseases.

### Clinical and Public Health Implications

The findings of this study have several potential implications for clinical practice and disaster response planning. Children with chronic allergic diseases should be considered a vulnerable population in post-disaster healthcare planning, and monitoring of disease control should be incorporated into follow-up programs in affected regions. Environmental control strategies in temporary settlements—including improved ventilation, reduction in indoor allergen exposure, and improved housing conditions—may help reduce respiratory irritants. In addition, dust-control measures during demolition and reconstruction activities may reduce airborne particulate exposure, particularly in residential areas and around schools. Strengthening air-quality monitoring systems and integrating environmental health surveillance into disaster management strategies may help identify high-risk populations and support preventive interventions for children with chronic allergic diseases. Future prospective and multicenter studies are needed to further clarify the long-term health effects of environmental exposures following disasters and to inform strategies aimed at protecting vulnerable pediatric populations.

## Figures and Tables

**Table 1:** Baseline Characteristics of the Study Population (T0).

Variable	Total (n = 528)	Asthma (n = 212)	Allergic Rhinitis (n = 164)	Atopic Dermatitis (n = 152)
Age (years), mean ± SD	8.7 ± 4.2	9.1 ± 4.0	9.4 ± 4.1	6.8 ± 3.8
Male sex, n (%)	302 (57.2)	121 (57.1)	92 (56.1)	89 (58.6)
Disease duration (years), mean ± SD	3.1 ± 2.2	3.6 ± 2.3	3.2 ± 2.0	2.1 ± 1.7
Passive tobacco smoke exposure, n (%)	211 (39.9)	91 (42.9)	63 (38.4)	57 (37.5)
Indoor dampness or mold, n (%)	146 (27.6)	63 (29.7)	44 (26.8)	39 (25.7)

Values are presented as mean ± SD or n (%). SD: standard deviation.

**Table 2:** Post-Earthquake Environmental Exposure Characteristics.

Exposure Variable	n (%)
Temporary housing ≥ 6 months	187 (35.4)
Temporary housing < 6 months	118 (22.3)
No temporary housing	223 (42.2)
Demolition-related dust exposure	241 (45.6)
High particulate matter exposure area	198 (37.5)

Temporary housing included container or tent settlements. Dust exposure was defined based on caregiver-reported demolition or debris removal activities around the home or school.

**Table 3:** Changes in Disease-Specific Clinical Outcomes Between Baseline and Follow-Up.

Outcome	Baseline (T0)	Follow-Up (T1)	Change (Δ)	*p*-Value
ACT/cACT score	18.2 ± 4.6	16.5 ± 6.2	−1.7 ± 4.8	0.02
TNSS	3.4 ± 1.7	3.9 ± 2.8	+0.5 ± 2.1	0.06
SCORAD index	35.8 ± 19.8	43.1 ± 24.5	+7.3 ± 18.4	0.03
Annual exacerbations	0.9 ± 1.2	1.8 ± 2.1	+0.9 ± 1.8	<0.001

Values are presented as mean ± SD. ACT/cACT: Asthma Control Test/Childhood Asthma Control Test. TNSS: Total Nasal Symptom Score. SCORAD: Scoring Atopic Dermatitis.

**Table 4:** Multivariable Logistic Regression Analysis of Factors Associated With Clinical Deterioration.

Variable	Adjusted OR	95% CI	*p*-Value
Temporary housing ≥6 months	2.1	1.2–3.9	0.01
Demolition-related dust exposure	1.9	1.1–3.5	0.02
High particulate matter exposure	1.6	0.9–2.8	0.08
Passive tobacco smoke exposure	1.7	1.0–2.9	0.04
Indoor dampness or mold	1.4	0.8–2.5	0.22

The model included all listed exposure variables and was additionally adjusted for age, sex, socioeconomic status, baseline disease severity, and disease category. OR: odds ratio; CI: confidence interval.

**Table 5:** Multivariable Linear Regression Analysis of Factors Associated With Change in ACT/cACT Score in Children With Asthma.

Variable	Adjusted β	95% CI	*p*-Value
Temporary housing ≥6 months	−1.84	−3.02 to −0.66	0.002
Demolition-related dust exposure	−1.39	−2.54 to −0.24	0.018
High particulate matter exposure	−1.12	−2.31 to 0.07	0.065
Passive tobacco smoke exposure	−0.96	−2.01 to 0.09	0.072
Indoor dampness or mold	−0.71	−1.88 to 0.46	0.233

Dependent variable: change in ACT/cACT score between baseline and follow-up (T1 − T0). Negative β values indicate worsening asthma control. The model was adjusted for age, sex, baseline ACT/cACT score, passive tobacco smoke exposure, and indoor dampness.

**Table 6:** Mean Ambient Particulate Matter Levels in Malatya Before and After the Earthquake.

Parameter	Pre-Earthquake	Post-Earthquake	Relative Change
PM10 (µg/m^3^)	30.1	107.6	+257%
PM2.5 (µg/m^3^)	16.8	47.6	+183%

Data were obtained from the Turkish National Air Quality Monitoring Network. Values represent mean regional PM concentrations during the predefined pre-earthquake and post-earthquake monitoring periods.

## Data Availability

The data supporting the findings of this study are not publicly available due to privacy and ethical restrictions but are available from the corresponding author upon reasonable request.

## Supplementary Materials

The following supporting information can be downloaded at https://www.mdpi.com/article/10.3390/jcm15082875/s1, Table S1: Subgroup Analyses of Environmental Exposures and Clinical Deterioration.
