# Supplementary material for: Clinical Implications of Post-Earthquake Environmental Exposures in Children with Allergic Diseases
Source: J Clin Med. 2026 Apr 10;15(8):2875. doi: 10.3390/jcm15082875 (PMC13115785; doi:10.3390/jcm15082875)
Supplement: Supplementary file 1 [file jcm-15-02875-s001.zip › jcm-4229497-supplementary.pdf]

Journal of
Clinical Medicine

MDPI

Supplementary Table S1. Subgroup Analyses of Environmental Exposures and Clinical Deterioration.

Subgroup	Exposure	OR	95% CI	p interaction
Age 6–11	Temporary housing	2.6	1.1–6.2	0.18
Severe baseline disease	Demolition dust	2.9	1.3–6.7	0.03

OR: odds ratio; CI: confidence interval.
